# Efficacy of levomilnacipran extended-release in major depressive disorder: pooled analysis of 5 double-blind, placebo-controlled trials

**DOI:** 10.1017/S1092852914000273

**Published:** 2014-06-05

**Authors:** Stuart A. Montgomery, Carl P. Gommoll, Changzheng Chen, William M. Greenberg

**Affiliations:** 1 Imperial College School of Medicine, University of London, London, UK; 2 Clinical Development, Forest Research Institute, Jersey City, New Jersey, USA; 3 Biostatistics, Forest Research Institute, Jersey City, New Jersey, USA

**Keywords:** major depressive disorder, levomilnacipran, antidepressant, serotonin and norepinephrine reuptake inhibitor, response, remission

## Abstract

**Introduction/Objective:**

Post hoc analyses were conducted to evaluate the efficacy of levomilnacipran extended-release (ER) in subgroups of patients with major depressive disorder (MDD).

**Methods:**

Data were pooled from 5 completed Phase II/III studies. Patients were categorized by sex, age, MDD duration, recurrence of MDD, current episode duration, number of prior episodes, and baseline Montgomery–Åsberg Depression Rating Scale (MADRS) score. Efficacy was evaluated by MADRS least squares (LS) mean change from baseline, response (MADRS improvement ≥50%), and remission (MADRS ≤10).

**Results:**

In the pooled population, treatment with levomilnacipran ER versus placebo resulted in greater improvement in MADRS score (−15.8 versus −12.9; LS mean difference, −2.9; *P* < .001) and higher response rates (44.7% versus 34.5%; *P* < .001). Comparable treatment effects were found in most subgroups. Remission rates in the overall population were higher for levomilnacipran ER versus placebo (27.7% versus 21.5%; *P* < .05); notably high remission rates were seen in patients with baseline MADRS score < 30 (48.8% versus 28.9%; *P* < .001).

**Discussion:**

Clinically meaningful improvements in depressive symptoms were found across subgroups, including statistically significant outcomes for both response and remission.

**Conclusion:**

Levomilnacipran ER was efficacious across a wide range of MDD patients, including men and women, ages 18–78, with varying histories and symptom severity.

## Introduction

Patients with major depressive disorder (MDD) are a heterogeneous population, with different symptoms and varying degrees of disease severity. The worldwide occurrence of MDD varies by country, but epidemiologic data generally indicate that this disorder is both common and chronic.^1^ In the United States, the lifetime prevalence for MDD is estimated to be 16.2%,^2^ affecting all ethnic groups,^3^ ^–^ ^5^ and with onset generally occurring during early adulthood.^6^ Most patients have a chronic or recurrent course of illness,^7^ which negatively affects patient quality of life even during periods of remission.^8^

Many patients do not achieve response and/or remission following initial antidepressant therapy, but may do so with subsequent treatment regimens.^9^ No single pharmacologic therapy is effective in every individual. Identifying medications with efficacy across patient subgroups continues to be of clinical interest, and several studies have evaluated the effects of patient demographics,^10^ ^–^ ^13^ MDD history,^11^ ^,^ ^14^ ^–^ ^16^ and symptom severity^11^ ^,^ ^16^ ^,^ ^17^ on treatment outcomes. For some antidepressants, treatment outcomes can vary significantly based on patient characteristics: for example, male sex, older age, longer duration of illness, more chronic depression, greater number of prior episodes, and more severe depression at baseline have been associated with lower antidepressant efficacy and/or worse treatment outcomes.^9^ ^,^ ^10^ ^,^ ^14^ ^,^ ^18^ ^–^ ^21^

Levomilnacipran is a potent and selective serotonin and norepinephrine reuptake inhibitor (SNRI) that is approved for the treatment of MDD in adult patients.^22^ Levomilnacipran (1*S*, 2*R*-) is the more active enantiomer of the racemate, milnacipran, which is approved in the United States for the management of fibromyalgia.^23^ Preclinical studies with levomilnacipran have shown that it has higher potency for inhibiting the norepinephrine and serotonin transporters relative to the less active enantiomer, F2696 (1*R*, 2*S*-).^24^ Levomilnacipran also has a more favorable pharmacokinetic profile than F2696, with greater plasma concentrations and slower elimination. Additionally, in contrast to venlafaxine and duloxetine, which show greater preference for serotonin relative to norepinephrine reuptake inhibition, levomilnacipran has greater potency for inhibiting norepinephrine than serotonin reuptake.^24^ In terms of clinical efficacy, no valid comparisons can be made between levomilnacipran and milnacipran or other SNRIs, since head-to-head trials with these drugs have not been conducted.

An extended-release (ER) formulation of levomilnacipran was developed for once-daily dosing. The safety and efficacy of levomilnacipran ER in the treatment of MDD in adult patients was evaluated in 5 Phase II/III clinical studies.^25^ ^–^ ^29^ Levomilnacipran ER was generally safe and well tolerated in these studies. On the primary efficacy measure, mean change from baseline on the clinician-rated Montgomery–Åsberg Depression Rating Scale (MADRS), significantly greater reductions were seen for levomilnacipran ER relative to placebo in 2 fixed-dose studies (40, 80, or 120 mg/day^25^; 40 or 80 mg/day^26^) and 2 flexible-dose studies (40–120 mg/day^28^; 75–100 mg/day^29^). Significant differences from placebo were also seen on the secondary outcome measure, the Sheehan Disability Scale (SDS) total score, suggesting that levomilnacipran ER is effective in improving depression-related functional impairment. In the remaining study,^27^ which was another flexible-dose study (40–120 mg/day), the least squares mean differences (LSMDs) between levomilnacipran ER and placebo showed numerical advantages for MADRS and SDS, but the advantages did not reach statistical significance. To further evaluate the efficacy of levomilnacipran ER in MDD, data from all 5 studies were pooled and analyzed by patient subgroups.

## Methods

### Clinical studies

The analyses are based on pooled data from 5 randomized, double-blind, placebo-controlled, multicenter trials of levomilnacipran ER 40–120 mg/day in adults with MDD, which included 2 fixed-dose studies^25^ ^,^ ^26^ and 3 flexible-dose studies.^27^ ^–^ ^29^ One of the flexible-dose studies was a non-U.S. phase II trial (Europe, India, and South Africa)^29^; the other four studies were U.S. phase III studies.^25^ ^–^ ^28^ Study designs and methods were generally similar and have been reported in detail in the published trial results.

The levomilnacipran ER studies included female and male patients, ages 18–80 years, who met *Diagnostic and Statistical Manual of Mental Disorders*, fourth edition, text revision (DSM-IV-TR)^30^ criteria for MDD and current depressive major depressive episode, with one study^26^ requiring a history of recurrent episodes. Patients were also required to meet criteria based on MADRS, Clinician Global Improvement of Severity (CGI-S), 17-item Hamilton Depression Rating Scale (HAMD_17_), and/or SDS scores (Table 1). Those who had DSM-IV-TR Axis I disorders other than MDD, social anxiety disorder, generalized anxiety disorder, or specific phobia were excluded from the U.S. studies.^25^ ^–^ ^28^ In the non-U.S. study, patients with comorbid panic disorder, agoraphobia, obsessive compulsive disorder, generalized anxiety, posttraumatic stress disorder, or social phobia were excluded if onset preceded the current depressive episode.^29^ Other key exclusion criteria included history of nonresponse to ≥2 antidepressants after adequate treatment and significant risk of suicide based on investigator judgment or formal assessment, such as the Columbia-Suicide Severity Rating Scale (C-SSRS) or suicide-related items from MADRS, HAMD_17_, or the Mini-International Neuropsychiatric Interview.

**Table 1:** Summary of levomilnacipran extended-release clinical studies

	Study design	Inclusion criteria	Treatment groups (n)
U.S. Study 1^25^	1-week single-blind PBO run-in period;	MADRS score ≥30	PBO = 179
NCT00969709	8-week randomized, double-blind, fixed-dose treatment;	MADRS-SR score ≥26	LVM ER
	2-week double-blind taper		40 mg = 181
			80 mg = 181
			120 mg = 183
U.S. Study 2^26^	1-week single-blind PBO run-in period;	MADRS score ≥26	PBO = 189
NCT01377194	8-week randomized, double-blind, fixed-dose treatment;	CGI-S score ≥4	LVM ER
	1-week double-blind taper	Recurrent MDD	40 mg = 190
			80 mg = 189
U.S. Study 3^27^	1-week single-blind PBO run-in period;	MADRS score ≥30	PBO = 184
NCT00969150	8-week randomized, double-blind, flexible-dose treatment;	MADRS-SR score ≥26	LVM ER
	2-week double-blind taper		40–120 mg = 178
U.S. Study 4^28^	1-week single-blind PBO run-in period;	MADRS score ≥30	PBO = 220
NCT01034462	8-week randomized, double-blind, flexible-dose treatment;	MADRS-SR score ≥26	LVM ER
	2-week double-blind taper		40–120 mg = 222
Non-U.S. Study^29^	10-week randomized, double-blind, flexible-dose treatment;	HAMD_17_ score >22	PBO = 281
EudraCT: 2006-002404-34	1-week double-blind taper	SDS score ≥10	LVM-ER
		At least 1 SDS subscale score ≥6	75–100 mg = 282

Abbreviations: CGI-S, Clinician Global Improvement of Severity; HAMD_17_, 17-item Hamilton Depression Rating Scale; LVM ER, levomilnacipran extended-release; MADRS, Clinician-Rated Montgomery–Åsberg Depression Rating Scale; MADRS-SR, MADRS Self-Rated; PBO, placebo; SDS, Sheehan Disability Scale.

The primary efficacy measure in all studies was evaluated by LSMD versus placebo for change from baseline in MADRS total score at 8 (or 10) weeks. Additional prespecified measures included the percentage of patients who met criteria for response (defined as ≥50% improvement from baseline in MADRS total score) and remission (MADRS total score ≤10) at end of treatment.

### Post hoc analyses

To further explore the effects of patient characteristics on treatment outcomes, post hoc analyses were conducted on pooled data from participants in the levomilnacipran ER studies. For consistency with the individual studies, efficacy in the overall pooled population was evaluated based on the following analyses: (1) least squares (LS) mean changes from baseline to end of double-blind treatment (week 8 in U.S. studies^25^ ^–^ ^28^; week 10 in the non-U.S. study^29^) in MADRS total score, (2) treatment response (ie, ≥50% improvement in MADRS total score), and (3) disease remission (ie, MADRS total score ≤10) at the end of treatment. To further explore the clinical relevance of these outcomes, treatment effect sizes for MADRS total score improvements are provided, along with numbers needed to treat (NNT) for response and remission rates.

These analyses were also conducted in subgroups of patients, categorized by sex (male or female), age (18 to <45, ≥45 to <60, or ≥60 years), mean duration of illness (MDD diagnosis <2, ≥2 to <10, or ≥10 years), recurrent MDD (yes or no), duration of current MDD episode (<6, ≥6 to 12, or ≥12 months; U.S. studies only), number of prior episodes (1–2, 3–4, ≥5), depression severity (MADRS baseline score <30, ≥30, or ≥35).

### Statistical analyses

For the post hoc analyses presented in this report, the overall pooled population was defined as patients who received ≥1 dose of double-blind study drug and had ≥1 post-baseline MADRS assessment. Levomilnacipran ER dosages were pooled for comparison with placebo. Baseline characteristics were analyzed based on available data (ie, observed cases) in each subgroup. For efficacy analyses, comparisons between treatment groups were analyzed in each subgroup.

Analyses of LS mean change from baseline to the end of the double-blind treatment period were based on nonmissing data using a mixed-model for repeated measures (MMRM) with study, treatment group, pooled study center, visit, subgroup, treatment-by-subgroup, subgroup-by-visit, treatment group-by-visit, and subgroup-by-treatment-by-visit interactions as fixed effects and the baseline and baseline-by-visit interactions as the covariates. Treatment effect sizes for improvements in MADRS total score were estimated using Cohen's *d*. Response and remission rates were analyzed using a logistic regression model with the treatment group and baseline MADRS score as explanatory variables; missing values were imputed using a last observation carried forward (LOCF) approach. *P*-values were not adjusted for multiple comparisons. NNTs were calculated as the reciprocal of the difference between levomilnacipran ER and placebo for response and remission rates, with 95% confidence intervals (95% CIs) also calculated as the reciprocal of the intervals for the rate difference.

## Findings

The overall pooled population included 2598 patients (placebo, n = 1032; levomilnacipran ER, n = 1566). Baseline characteristics were similar between treatment groups (Table 2). The majority of the overall population was female (63.8%), white (79.9%), and < 60 years of age (89.8%). Most patients (83.0%) had been diagnosed with MDD for at least 2 years; 45.8% of patients reported a history of MDD for ≥10 years. The majority of patients (79.9%) also had a history of recurrent depressive episodes, with 63.4% of patients reporting 3 or more prior episodes. As was expected based on study entry criteria, > 80% of patients had a baseline MADRS total score ≥30, and approximately 40% of patients had a baseline MADRS total score ≥35.

**Table 2:** Patient baseline characteristics in the overall pooled population*

	Placebo		Levomilnacipran ER	
Characteristics	N	Value	N	Value
Female, n (%)	1032	660 (64.0)	1566	997 (63.7)
White, n (%)	1031	846 (82.1)	1566	1228 (78.4)
Age				
Mean age (SD), years	1032	43.5 (12.7)	1566	42.7 (12.9)
Age <45 years, n (%)	1032	518 (50.2)	1566	830 (53.0)^‡^
Age ≥45 to <60 years, n (%)	1032	408 (39.5)	1566	576 (36.8)
Age ≥60 years, n (%)	1032	106 (10.3)	1566	160 (10.2)
MDD history				
Mean age at onset (SD), years	1030	32.0 (13.8)	1565	31.4 (13.5)
Mean duration (SD), years	1030	11.4 (11.0)	1565	11.3 (10.8)
Duration <2 years, n (%)	1030	186 (18.1)	1565	254 (16.2)
Duration ≥2 to <10 years, n (%)	1030	378 (36.7)	1565	588 (37.6)
Duration ≥10 years, n (%)	1030	466 (45.2)	1565	723 (46.2)
With recurrent MDD, n (%)	949	772 (81.3)	1503	1186 (78.9)
Current MDD episode^†^				
Duration <6 months, n (%)	754	318 (42.2)	1290	531 (41.2)
Duration ≥6 to <12 months, n (%)	754	239 (31.7)	1290	441 (34.2)
Duration ≥12 months, n (%)	754	197 (26.1)	1290	318 (24.7)
Prior MDD episodes				
Mean number of episodes (SD)	838	4.2 (4.4)	1269	4.1 (5.6)
1 to 2 episodes, n (%)	772	272 (35.2)	1186	444 (37.4)
3 to 4 episodes, n (%)	772	288 (37.3)	1186	452 (38.1)
≥5 episodes, n (%)	772	212 (27.5)	1186	290 (24.5)
Baseline MADRS score				
Mean total score (SD)	1032	33.3 (4.6)	1566	33.8 (4.5)
Total score <30, n (%)	1032	190 (18.4)	1566	250 (16.0)
Total score ≥30, n (%)	1032	842 (81.6)	1566	1316 (84.0)
Total score ≥35, n (%)	1032	380 (36.8)	1566	629 (40.2)

The values in parentheses indicate standard deviation or percent of patients, as indicated for each characteristic.

* For the intent-to-treat population, defined as all patients who received ≥1 dose of study drug and had ≥1 postbaseline MADRS assessment; N = number of patients with available data.

† Data not collected in the non-U.S. study.

‡ One patient from this group did not attend any scheduled study visits and is excluded from the efficacy analyses.

Abbreviations: ER, extended-release; MADRS, Clinician-Rated Montgomery–Åsberg Depression Rating Scale; MDD, major depressive disorder; SD, standard deviation.

**Figure 1:**
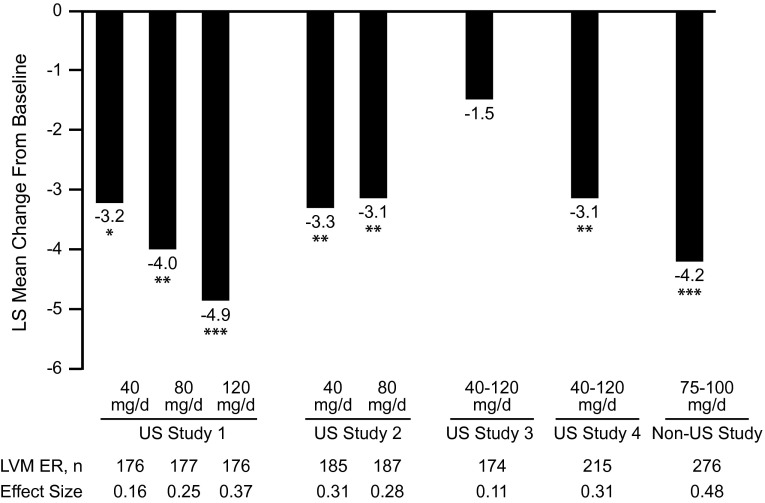
Primary efficacy outcomes in levomilnacipran ER studies. Least squares mean differences between treatment arms in changes from baseline in MADRS total score. *P < 0.05; **P < 0.01; ***P < 0.001 versus placebo. Abbreviations: LS, least squares; LVM ER, levomilnacipran extended-release; MADRS, Montgomery–Åsberg Depression Rating Scale; PBO, placebo.

Significantly greater improvements from baseline on the predefined primary outcome (ie, MADRS total score) were seen with levomilnacipran ER compared with placebo in 4 of 5 studies (Figure 1). The LSMDs between levomilnacipran ER and placebo were statistically significant in 2 fixed-dose studies (range, −3.1 to –4.9; *P* < .05)^25^ ^,^ ^26^ and 2 flexible-dose studies (range, −3.1 to −4.2; *P* < .05).^28^ ^,^ ^29^ In 1 flexible-dose study, the LSMD from placebo did not reach statistical significance (−1.5; *P* = .25).^27^ Calculated treatment effect sizes ranged from 0.16–0.48 in the positive studies,^25^ ^,^ ^26^ ^,^ ^28^ ^,^ ^29^ with no clear pattern of a dose-related response (Figure 1).

Improvements in MADRS total score were also significantly greater with levomilnacipran ER than placebo in the overall pooled population and in all patient subgroups, except for those with MDD duration <2 years or current episode duration ≥12 months (Table 3), which showed a numerical advantage for levomilnacipran ER but did not reach statistical significance. The LSMD between active treatment and placebo was −3.0 (*P* < .001) in the overall population. A comparable magnitude of treatment effect was found in all subgroups (range, −2.1 to −4.4) except for patients with MDD duration < 2 years, which showed slightly lower treatment effects (−1.7). A possible interaction was detected between treatment and gender (*P* = .09); no interactions were found with other baseline factors (all *P* > .3).

**Table 3:** Least squares mean changes from baseline in MADRS total score in the overall pooled study population and patient subgroups

	Placebo		Levomilnacipran ER			
Population or subgroup	n	LSM change (SE)	n	LSM change (SE)	LSM difference (95% CI)	*P*-value
Overall pooled population	1032	−12.9 (0.4)	1566	−15.8 (0.3)	−3.0 (−3.9, −2.1)	< 0.001
Sex						
Female	660	−12.9 (0.4)	997	−15.3 (0.4)	−2.4 (−3.5, −1.3)	< 0.001
Male	372	−12.8 (0.6)	569	−16.9 (0.5)	−4.0 (−5.6, −2.5)	< 0.001
Age						
< 45 years	518	−13.4 (0.5)	829	−16.1 (0.4)	−2.7 (−4.0, −1.4)	< 0.001
≥ 45 to < 60 years	408	−12.3 (0.6)	576	−15.3 (0.5)	−2.9 (−4.4, −1.5)	< 0.001
≥ 60 years	106	−12.3 (1.1)	160	−16.7 (0.9)	−4.4 (−7.2, −1.6)	0.002
MDD duration						
< 2 years	186	−14.4 (0.8)	254	−16.1 (0.8)	−1.7 (−3.9, 0.5)	0.129
≥ 2 to < 10 years	378	−13.0 (0.6)	587	−15.9 (0.5)	−2.9 (−4.4, −1.3)	< 0.001
≥ 10 years	466	−12.1 (0.5)	723	−15.7 (0.4)	−3.6 (−4.9, −2.2)	< 0.001
Recurrent MDD						
Yes	772	−12.4 (0.4)	1185	−15.7 (0.4)	−3.3 (−4.3, −2.2)	< 0.001
No	177	−13.4 (0.9)	317	−16.0 (0.7)	−2.6 (−4.8, −0.4)	0.019
Duration of current episode						
< 6 months	318	−12.1 (0.7)	531	−15.6 (0.5)	−3.5 (−5.1, −1.8)	< 0.001
≥ 6 to < 12 months	239	−12.7 (0.8)	441	−15.6 (0.6)	−2.9 (−4.7, −1.0)	0.003
Duration ≥ 12 months	197	−11.9 (0.9)	317	−14.0 (0.7)	−2.1 (−4.2, 0.0)	0.054
Number of prior episodes						
1 to 2	272	−12.6 (0.7)	444	−16.0 (0.6)	−3.4 (−5.2, −1.7)	< 0.001
3 to 4	288	−12.9 (0.7)	452	−16.0 (0.6)	−3.1 (−4.8, −1.4)	< 0.001
≥ 5	212	−11.9 (0.8)	290	−14.9 (0.7)	−3.0 (−5.1, −0.9)	0.004
Baseline MADRS total score						
< 30	190	−12.4 (0.9)	250	−15.9 (0.8)	−3.5 (−5.6, −1.3)	0.002
≥ 30	842	−13.0 (0.4)	1315	−15.8 (0.3)	−2.9 (−3.9, −1.9)	< 0.001
≥ 35	380	−12.9 (0.7)	629	−16.1 (0.6)	−3.2 (−4.7, −1.7)	< 0.001

Abbreviations: CI, confidence interval; LSM, least squares mean; MADRS, Clinician-Rated Montgomery–Åsberg Depression Rating Scale; SE, standard error.

**Figure 2:**
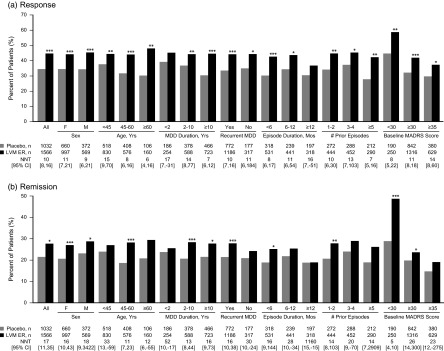
MADRS response and remission rates in the overall pooled study population and patient subgroups. Response defined as ≥50% improvement from baseline in MADRS total score. Remission defined as MADRS total score ≤10 at study endpoint. *P < 0.05; **P < 0.01; ***P < 0.001 versus placebo. Abbreviations: CI, confidence interval; LVM ER, levomilnacipran extended-release; MADRS, Montgomery–Åsberg Depression Rating Scale; NNT, number needed to treat; PBO, placebo.

The percentage of patients meeting the MADRS criterion for treatment response was higher with levomilnacipran ER than with placebo (Figure 2A). In the overall population, the difference between levomilnacipran ER and placebo response rates was 10.2% (*P* < .001); similar advantages for levomilnacipran versus placebo were found in most of the patient subgroups. Response rates were notably high for levomilnacipran relative to placebo in patients who were ≥60 years old (17.9% difference), reported ≥5 prior depressive episodes (14.6% difference), or had a baseline MADRS total score <30 (14.1% difference) (all *P* < .01). Significantly greater rates of response for levomilnacipran ER compared with placebo were found in all subgroups except for patients with MDD duration <2 years or current episode duration ≥12 months (as was observed for LS mean change in MADRS total score). In the overall pooled population, the NNT (95% CI) for response was 10 (8, 16). NNTs were ≤10 in 9 subgroups, 11–15 in 8 subgroups, and 16–17 in the remaining 2 subgroups (Figure 2A).

The difference between levomilnacipran ER and placebo in remission rates was 6.2% (*P* < .05) in the overall population; similar advantages for levomilnacipran ER versus placebo were generally observed across most patient subgroups (Figure 2B). Differences in remission rates between active treatment and placebo were relatively high in patients with baseline MADRS score <30 (19.9% difference; *P* < .001) and relatively low in the subgroups with MDD duration <2 years (1.9% difference) and current episode duration ≥12 months (0.1% difference) (both *P* > .05). The NNT (95% CI) for remission in the overall population was 17 (11, 35). NNTs of 11–20 were found in 11 subgroups, and a NNT of 5 was found in patients with baseline MADRS total score <30. NNTs in the remaining subgroups varied widely (Figure 2B).

## Discussion

The analyses of pooled data from 5 randomized, placebo-controlled, double-blind studies demonstrate the efficacy of levomilnacipran ER across various subgroups of adult MDD patients. The primary efficacy measure in these studies was LS mean change from baseline in the clinician-rated MADRS. In the 4 studies that attained statistical significance, the LSMD between levomilnacipran ER and placebo was >3 points in each active treatment arm,^25^ ^,^ ^26^ ^,^ ^28^ ^,^ ^29^ which is greater than the 2-point difference that has been used to identify clinically relevant treatment effects for between-group comparisons in MDD patients.^31^ In order to identify clinically meaningful effects among individual patients, post hoc analyses of data from these trials were conducted using accepted definitions of response and remission that require more stringent thresholds of improvement.^32^

The primary results from the individual studies were supported by post hoc analyses conducted in the overall pooled population and in subgroups of patients stratified by sex, age, MDD duration, recurrent MDD, duration of current episode, number of prior episodes, and baseline MADRS total score. In the post hoc analyses, LSMDs between levomilnacipran ER and placebo were greater than the threshold that has been used to determine clinically meaningful improvement (>2 points),^31^ with statistical significance in the overall pooled population (−2.74, *P* < .001) and across most subgroups; these results further support the clinical relevance of the individual levomilnacipran ER trial data. Clinically relevant results were also found for treatment response, defined as the percentage of patients who had ≥50% improvement from baseline in MADRS total score. For the MADRS response analysis, which is a benchmark used by the European Medicines Agency to evaluate treatment outcomes in patients with MDD, a 10% difference from placebo is often considered to be clinically meaningful.^31^ This difference was found in the pooled study population (10.2%, *P* < .001) and in the majority of subgroups. NNTs for response ranged from 6 (age ≥60 years) to 17 (MDD duration <2 years), with approximately half of the subgroups (9 of 19) having a NNT ≤10.

One advantage of pooling data from 5 studies was that it provided a robust sample of men (n = 941) in which to evaluate the effects of levomilnacipran ER on depressive symptoms. This subgroup was of particular interest because of the lower prevalence of MDD in men than women^2^ and potentially lower response to antidepressant treatment in men.^33^ Analyses by sex indicated that levomilnacipran ER was at least as effective in men as in women. The adjusted mean difference from placebo in MADRS total score change was greater in men than in women (−4.0 versus −2.4), as was the difference from placebo in treatment response rates (11.1% versus 9.7%), and was statistically significant (*P* < .001) in both men and women. For remission, however, the difference between active treatment and placebo was slightly higher in women than men (6.5% versus 5.7%; *P* < .05 in each subgroup). These subgroup analyses indicate that levomilnacipran ER is effective in both men and women.

Some studies have suggested that antidepressants may be less effective in older MDD patients.^19^ ^,^ ^34^ To analyze efficacy by age, patients were stratified using cutoffs from the National Comorbidity Survey Replication data (ie, <45, ≥45 to <60, and ≥60 years).^2^ Significant and clinically relevant effects with levomilnacipran ER were found in all of the age subgroups, with patients ≥60 years old having the greatest LSMD in MADRS total score change (−4.4; *P* = .002) and the highest difference from placebo in response rates (17.9%, *P* < .01). These findings are worth noting, given the inconsistent treatment effects that have been found with second-generation antidepressants in older patients, as reported in a meta-analysis of 10 randomized, placebo-controlled trials in older (ie, ≥60 years) MDD outpatients.^34^ Although the meta-analysis found a significant difference between active drug and placebo in pooled response rates (9.7% difference; odds ratio = 1.40; *P* < .001), results of the individual studies ranged from <0% drug–placebo difference (ie, greater effect with placebo than active drug) to 21% drug–placebo difference, with 4 of the 10 trials reporting nonsignificant findings. In addition to demonstrating significant treatment response, older patients in the levomilnacipran ER studies had remission rates (difference from placebo, 8.6%) that were comparable to patients ages ≥45 to <60 years (difference from placebo, 9.7%; *P* < .001) and higher than in patients <45 years of age (difference from placebo, 3.1%). Overall, these results indicate that levomilnacipran ER is efficacious in older as well as younger adult patients, which is an important finding given the historical difficulty of establishing efficacy in patients who are ≥60 years of age.

Although the individual levomilnacipran ER studies were not powered to detect statistical between-group differences in remission, and the 8- to 10-week treatment duration may not have been sufficient for some patients to achieve this outcome, significant results for remission were found nonetheless in 2 of the studies.^25^ ^,^ ^29^ Pooling the individual study data provided an opportunity to further explore the effects of levomilnacipran ER in various patient subgroups using MADRS total score ≤10 as the criterion for remission. The difference in remission rates between levomilnacipran ER and placebo was significant in the overall pooled population (6.2%; *P* < .05), and the numerical differences consistently favored levomilnacipran ER over placebo in almost all patient subgroups. The NNTs for remission ranged widely across subgroups, although the majority of subgroups (11 of 19) had NNTs of 10 to 20. However, some caution should be taken when interpreting these results, since pooling the data may have increased the potential bias for signal detection without accounting for the variability of results found among the individual studies.

The high percentage of patients (> 80%) with baseline MADRS total score ≥30, indicating considerable symptom burden,^35^ was primarily due to entry criteria used in many of the U.S. studies.^25^ ^–^ ^28^ Clinically relevant and statistically significant improvements with levomilnacipran ER relative to placebo were found in these patients, which included the subgroups with baseline MADRS total score ≥30 or ≥35. Both of these subgroups had comparable advantages over placebo in mean MADRS score improvements (LSMDs, −2.9 and −3.2, respectively; both *P* < .001), response rates (differences versus placebo, 9.8% and 7.7%, respectively; both *P* < .05), and remission rates (differences versus placebo, 3.9% and 4.4%, respectively; *P* < .05 for MADRS ≥30). However, since patients from the non-U.S. study^29^ were also included in the post hoc analyses, treatment effects with levomilnacipran ER could also be evaluated in patients with less severe depressive symptoms (MADRS score < 30). Although it has been suggested that antidepressants may be less effective in such patients,^36^ clinically meaningful treatment effects with levomilnacipran ER were found in the MADRS < 30 subgroup, as indicated by mean improvements in MADRS total score (LSMD, −3.5; *P* < .01) and the significant differences from placebo for both response (14.1%, *P* < .01) and remission (19.9%, *P* < .001), corresponding to NNTs of 8 and 5, respectively. Overall, these results from the MADRS subgroup analyses suggest that levomilnacipran ER may be efficacious in patients with varying levels of symptom severity.

The only 2 subgroups that did not have statistically significant mean improvements or treatment response to levomilnacipran ER relative to placebo were those with current episode duration ≥12 months or MDD duration <2 years. In the subgroup of patients with a current episode ≥12 months, the response rates for both levomilnacipran ER and placebo were lower than in the subgroup with shorter current episodes. However, these findings should be interpreted with caution, as it is possible that the longer duration subgroup included patients with more chronic and possibly refractory MDD who were less likely to respond to any antidepressant treatment. In the subgroup of patients with MDD duration <2 years, the nonsignificant results were somewhat surprising, since it seems likely that these patients would have had less severe depression than patients with longer duration. However, the results in Table 3 and Figure 2 suggest that this group of patients may be more responsive to placebo effects, thereby reducing assay sensitivity to detect active treatment benefits. Additionally, this subgroup was relatively small, which may have limited the sensitivity to detect significant treatment differences.

One limitation of these post hoc analyses is that the levomilnacipran ER clinical trials were not specifically designed to stratify patients by sex, age, or any other baseline factors. Although most of these categories were large enough to detect statistically significant treatment effects, the subgroup sizes did range from 266 patients (age ≥60 years) to 2157 patients (baseline MADRS score ≥30), and some subgroups may have been too small to detect statistical differences between treatment arms. In addition, the individual studies varied in dosage, geographic location, and inclusion criteria. Other potential limitations have already been discussed, including short duration of treatment and the exclusion of patients with baseline MADRS total score < 30 in 3 U.S. studies. In addition, significance testing was not adjusted for multiple comparisons. Nonetheless, the post hoc analyses presented in this report were based on data from large, well-controlled, Phase II/III studies that used the standardized and validated MADRS to evaluate the effects of active treatment versus placebo. Finally, it should be noted that although efficacy across subgroups has been the focus of this report, clinicians should also consider the safety and tolerability of levomilnacipran ER when treating patients. In general, this medication has been found to be well tolerated in adults with MDD; full details of the tolerability profile are available in the primary study results^25^ ^–^ ^29^ and in the current prescribing information.^22^

## Conclusion

The clinical trial data from U.S. and non-U.S. study sites indicate that levomilnacipran ER is an efficacious treatment in adult patients with MDD, with subgroup analyses suggesting meaningful improvements in the diverse patient populations seen in clinical practice, including both men and women, older and younger patients, and those with varying MDD histories and symptom severities.

## Disclosures

Stuart Montgomery has the following disclosures: Alkermes, consultant/speaker, consulting fee; AstraZeneca, consultant/speaker, consulting fee; Lundbeck, consultant/speaker, consulting fee; Pfizer, consultant/speaker, consulting fee; Pierre Fabre Médicament, consultant/speaker, consulting fee; Servier, consultant/speaker, consulting fee; Forest Labs, consultant/speaker, financial compensation was not received. Carl Gommoll, Changzheng Chen, and William Greenberg are employees of Forest Research Institute.
